# Paradoxical roles of autophagy in different stages of tumorigenesis: protector for normal or cancer cells

**DOI:** 10.1186/2045-3701-3-35

**Published:** 2013-09-09

**Authors:** Kai Sun, Weijie Deng, Shanshan Zhang, Ning Cai, Shufan Jiao, Jianrui Song, Lixin Wei

**Affiliations:** 1 Medical Sciences Research Center, Renji hospital, School of Medicine, Shanghai Jiaotong University, Shanghai, China; 2 Tumor Immunology and Gene Therapy Center, Eastern Hepatobiliary Surgery Hospital, The Second Military Medical University, Shanghai, China

**Keywords:** Autophagy, Protector, Early tumorigenesis, Tumor progression, Metastasis

## Abstract

Autophagy serves as a dynamic degradation and recycling system that provides biological materials and energy in response to stress. The role of autophagy in tumor development is complex. Various studies suggest that autophagy mainly contributes to tumor suppression during the early stage of tumorigenesis and tumor promotion during the late stage of tumorigenesis. During the tumorization of normal cells, autophagy protects genomic stability by retarding stem cells-involved damage/repair cycle, and inhibits the formation of chronic inflammatory microenvironment, thus protecting normal cell homeostasis and preventing tumor generation. On the other hand, autophagy also protects tumor cells survival during malignant progression by supporting cellular metabolic demands, decreasing metabolic damage and supporting anoikis resistance and dormancy. Taken together, autophagy appears to play a role as a protector for either normal or tumor cells during the early or late stage of tumorigenesis, respectively. The process of tumorigenesis perhaps needs to undergo twice autophagy-associated screening. The normal cells that have lower autophagy capacity are prone to tumorization, and the incipient tumor cells that have higher autophagy capacity possibly are easier to survival in the hash microenvironment and accumulate more mutations to promote malignant progression.

## Introduction

Currently, cancer is the leading cause of death in developed countries and the second in developing countries [1]. Further deepening the understanding of tumorigenesis is critical to the struggle against this severe public health problem. Tumorigenesis involves many fundamental alterations in basic cellular mechanisms and interactions with microenvironment. Genetic and epigenetic changes which results from the stimulations of numerous stresses initiate and promote the transformation from normal cells to benign tumor cells, and ultimately to malignant tumor cells [2]. Recent studies suggest that macroautophagy (hereafter as autophagy), as a homeostatic process, impacts on many cancer-associated factors and has various important functions in tumor development [3]. Many studies demonstrate that autophagy contributes to the adaptation of tumor cells to harsh microenvironment and chemotherapy. Targeting autophagy is considered as a promising therapeutic strategy in clinical cancer treatment. However, other studies show that autophagy deficiency results in various spontaneous tumors in mouse models. Autophagy seemingly plays dual roles as both tumor promotor and suppressor in tumorigenesis. This dynamic role of autophagy in tumor development appears mainly depend on tumor stage [4]. However, from another perspective, autophagy is always a protector in the process of tumor development. Autophagy appears protects normal cells or tumor cells during the early or late stage of tumorigenesis, respectively.

## Autophagy: process and basic functions

Autophagy is an evolutionarily conserved garbage elimination and internal recycling mechanism. The process of autophagy begins with the autophagosome formation which involves the segregation of cargo macromolecules in the double-membrane vesicle. Then autophagosomes fuse with lysosomes to form autolysosomes, where the cargos are degraded by lysosomal enzymes [5].

**Figure 1:**
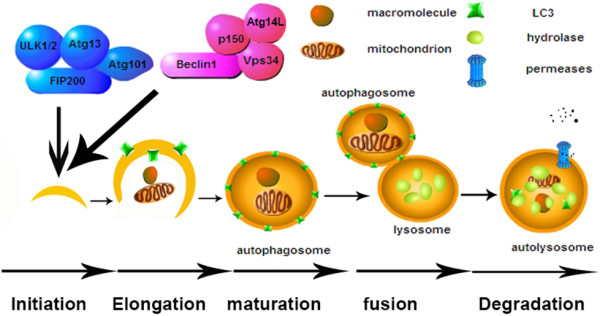
**Brief process of autophagy.** The process of autophagy includes five steps: initiation, elongation, maturation, fusion and degradation. Firstly, the cargos, which mainly include macromolecules and organelles, are encompassed by a double-membrane vesicle that gradually extends and ultimately forms autophagosome. Then autophagosome fuses with lysosome to form autolysosome, where the cargos are degraded by lysosomal hydrolase and the productions are recycled back to cytoplasm by lysosomal permease. ULK complex, which includes ULK1/2, Atg13, FIP200 and Atg101, is core machinery in initiation stage of autophagy. Beclin1-Vps34-Atg14L-p150 complex is the other key complex, which is required for Autophagosomal nucleation.

This complex process could be detailed divided into five steps, including initiation, elongation, maturation, fusion and degradation, which are controlled by a set of products of autophagy-related genes (Atgs) (Figure 1). Atgs were originally identified in yeast and partly have been identified the mammalian orthologs [6]. The core machinery of initiation stage is the Unc-51-like kinase (ULK) complex consisting of ULK, Atg13, FIP200 and Atg101 [7,8]. Changes in ULK1 (dephosphorylation and autophosphorylation) and dephosphorylation of Atg13 trigger the whole autophagic cascade [9]. Mammalian target of rapamycin (mTOR) kinase is one of the key negative regulators of autophagy induction. Activation of mTOR complex 1 (mTORC1) inhibits autophagy by inactivating ULK1/2 and Atg13 [10,11]. The activated ULK1 could further activate the other important complex, Beclin1-Vps34-Atg14L-p150 complex, by phosphorylating Beclin1 [12,13]. Activation of Beclin1 complex generates phosphatidylinositol-3-phosphate, which is the essential for nucleation of autophagic vesicles [12]. Autophagosome elongation and maturation involves two ubiquitin-like conjugation systems: microtubule-associated protein 1 light chain 3 (LC3) system and Atg12 system [14]. Phosphatidylethanolamine (PE) is conjugated to LC3I by Atg7 (E1 enzyme) and Atg3 (E2 enzyme). PE-conjugated LC3 becomes into a nonsoluble form (LC3-II) that stably inserted into the autophagosomal membrane [15]. Atg12 is conjugated to Atg5 by Atg7 and Atg10 (E2 enzyme). The Atg12-Atg5 heterodimer interacts with Atg16L, and then this complex promotes the elongation of autophagic membrane [16]. In addition, cargo selection is partly achieved by targeted ubiquitination, which is recognized by ubiquitin-interacting domains of autophagic cargo receptor proteins such as p62/SQSTM1. And then the cargo is targeted to autophagosome via LC3-interacting regions of p62 [17]. The fusion of autophagosomes, including their fusion with early and late endosomes, and lysosomes, is regulated by many molecules including Rubicon, UVRAG, Rab7, SNAREs, LAMPs [18-22]. In the final stage, the cargos are degraded by lysosomal hydrolase in the autolysosomes, and the productions are transported back to cytosol by lysosomal permease [5].

Autophagy plays an important role in the mechanism of keeping cellular homeostasis and survival, which could degrade damaged proteins and organelles to prevent toxic substances accumulation and recycle their components to regenerate metabolic precursors [23]. Under normal circumstances, autophagy maintains at a basal level to serve its housekeeping function. Besides this, autophagy also is induced as a self-protective response under many different forms of stress, such as nutrient and growth factor deprivation, hypoxia, microbe infection and so on [24,25]. Meanwhile, autophagy participates in the regulation of immune-system function and the suppression of inflammation [26]. Therefore, autophagy has close association with many diseases development including cancer [27,28].

## Autophagy protects normal cells against tumorization

### Autophagy deficiency promotes tumor generation

The first direct evidence of the relationship between autophagy and cancer was established in 1999, when Levine *et al.* discovered that Beclin1 is a candidate tumor suppressor gene. They found that Beclin1 is monoallelically deleted in a high percentage of human breast cancers and ovarian cancers, and Beclin1 expression is frequently low in human breast cancer, including cell lines and cancer tissues. Moreover, increase of Beclin1 expression in breast cancer cell lines inhibits cell proliferation *in vitro* and tumor generation in nude mice [29]. Then the importance of single copy loss of Beclin1 gene was exhibited in Beclin1 heterozygous knock-out mice, which is prone to develop spontaneous lymphomas, lung cancers, and liver cancers, and accelerates hepatitis B virus-induced hepatocarcinogenesis [30]. Levine *et al.* further found that Akt suppresses autophagy by mTOR-independent phosphorylation of Beclin1 and ultimately promotes tumorigenesis [31].

Besides Beclin1, many other components of autophagy machinery also play tumor-suppressive roles in tumorigenesis. Atg4C knock-out mice showed an increased susceptibility to develop carcinogen–induced fibrosarcomas [32]. Components of Beclin1/class III PI3K complex, UVRAG and Bif-1, also participate in the control of cell proliferation and suppression of tumorigenesis [18,33]. Notably, Atg5 mosaic deleted mice developed spontaneous benign liver tumors, but had no tumor detected in other organs. Liver-specific deletion of Atg7 also leads to benign liver tumors in the mice model [34]. These reports suggest that tumor suppression may be a property of whole autophagy machinery but not a signal autophagy protein. Meanwhile, total lack of autophagy may be a trigger of primary tumorigenesis, but not for malignant progression of late tumorigenesis. Although Beclin1 heterozygous knock-out mice also developed malignant tumors, the cells from Beclin1 heterozygous mice, in fact, still have palpable autophagic activity [4].

### Autophagy deficiency aggravates genome instability by accelerating stem cells-involved damage/repair cycle

How autophagy suppresses early tumorigenesis is an important question. In the ten hallmarks of cancer, genome instability is an enabling characteristic that has close association with the acquisition of other hallmark capabilities [2]. Many studies suggested that protecting genome stability is the crucial mechanism by which autophagy protects normal cells from tumorization.

Metabolic stresses distinctly impact on cellular genome stability. Metabolic stresses disturbs the mechanisms of DNA synthesis and repair by accumulating misfolded and aggregate-prone proteins, and triggers oxidative stress through accumulating reactive oxygen species (ROS)-generating organelles, especially mitochondria. In autophagy-competent cells, autophagy clears these accumulations to limit this metabolic stresses. Defective autophagy sensitizes normal cells to metabolic stress, and results in the increase of DNA damage, gene amplification and aneuploidy, and ultimately promotes tumorigenesis [35,36]. Further report showed that p62 protein plays an important role in the autophagy mediated-mitigation of metabolic stress. In autophagy-defective cells, aberrant p62 accumulation leads to cytotoxic response, such as activation of DNA damage response, change of gene expression, and elevation of chromosome instability that may accelerate DNA alterations and thereby contribute to tumorigenesis. Deregulation of NF-κB pathway and ROS accumulation involve in this process [37].

The study about microtubule-associated protein 1 small form (MAP1S) also suggested that dysfunctional autophagy is related to genome instability and tumorigenesis. MAP1S serves as a linker to bridge the autophagy machinery with microtubules and mitochondria to affect autophagosomal biosynthesis and degradation. In the murine model of diethylnitrosamine (DEN)-induced hepatocarcinogenesis, MAP1S elevation in response to DEN treatment enhances autophagy to remove p62-tagged misfolded proteins and damaged organelles that trigger DNA double-strand breaks and genome instability [38].

Although these studies demonstrated that autophagy plays an important role in the protection of genomic stability, the exact mechanism of how autophagy deficiency results in genomic instability has not been clarified. On the one hand, tumorigenesis is a multistep process, but mature cells usually have very limited proliferation ability. And the genomic maintenance systems can detect and repair the defects in the DNA, which ensures that the incidence of genomic mutation is often very low during each cell generation [2]. On the other hand, vast accumulation of cell damage often leads to cell apoptosis, necrosis or senescence, all of which are the barriers of tumorization of normal cells. There is a question that how autophagy-deficiency cells keep alive and undergo enough cell generations, and ultimately accumulate various DNA mutation which results in genomic instability.

We propose a hypothesis for this question. Early tumorigenesis, if which is not mediated by activated oncogene, often is a process of chronic tissue injury that contains a cycle of cell death and death-driven compensatory proliferation [39]. In this process, defective autophagy promotes cell death by enhancing accumulation of cell damage, and thus accelerates tissue damage/repair cycle. Continuous damage/repair cycle triggers activation of stem cells to support full regeneration. Autophagy deficiency also enhances stem cells injury. Stem cells with DNA defects and their daughter cells were repeatedly screened by outer and inner stresses. The cells with survival-promoting DNA mutation can alive in the process of chronic tissue injury, further accumulates non-lethal DNA defects, and ultimately has genomic instability.

The study about Atg5 mosaic deleted mice showed that tumors are only generated in the liver but not other organs [34]. The reason may be that compared to other organ, liver as the major metabolic organ has the most metabolic stresses, which brings about the fastest damage/repair cycle under the damage caused by autophagy deficiency. In another mice model, liver-specific Atg7-deficiency also promoted the death of liver cells [40]. Meanwhile, the studies about Atg7 and FIP200 indicated that autophagy deficiency lead to differentiation disorder and abnormal proliferation of stem cells, both of which may be the early events in the process of tumorigenesis [41,42]. These studies suggested that accelerating damage/repair cycle, especially the cycle involved stem cell activation, may play an important role in the autophagy-deficiency induced genomic instability.

### Autophagy deficiency promotes the formation of chronic inflammatory microenvironment

Chronic inflammation also is considered an enabling characteristic in the hallmarks of cancer. Inflammatory microenvironment can supply survival factors, growth factors, pro-angiogenic factors and other hallmark-facilitating factors to promote the acquisition of many core hallmark capabilities [2,43]. Many reports show that autophagy contributes to tumor suppression by inhibiting inflammation.

Necrotic cell death is an important factor for inducing inflammation, which releases various pro-inflammatory factors into the surrounding tissue microenvironment. Genetic inhibition of autophagy in apoptosis-defective immortalized epithelial cells leads to cell necrosis, chronic inflammation and ultimate tumorigenesis [44]. In α-1-antitrypsin-mutant mouse, autophagy inhibits liver carcinogenesis through disposing aggregation-prone protein and suppressing subsequent liver injury and inflammation [45]. In these reports, elevation of inflammation in autophagy-deficiency tissue may be related to the reduplicative cell necrosis in the liver. Release of IL-α by cell necrosis activates kupffer cells to produce cytokines, including TNF-α, IL-6 and hepatocyte growth factor, which promote activation of NF-κB pathway, compensatory proliferation and ultimate hepatocarcinogenesis [46]. Our previous study also showed that autophagy inhibition lead to the elevation of TNF-α, IL-1β and IL-6 expressions during DEN-induced early hepatocarcinogenesis [47].

On the other hand, autophagy also directly regulates the production of inflammatory signals. Autophagy-deficiency activates the inflammasome, which promotes the maturation of inflammatory cytokines, such as IL-1β and IL-18 [48]. Further researches show that mitochondrial reactive oxygen species (mtROS) produced by damaged mitochondria plays a crucial role in this process. Loss of autophagy function results in ROS-generating mitochondria accumulation. And ROS brings about activation of inflammasome NLRP3, which promotes the maturation of caspase1. Activated caspase1 cleaves pro-IL-1β to produce a matured IL-1β that is subsequently secreted from the cells [49]. In addition, mtROS also acts as a signaling molecule to trigger other inflammatory cytokines, such as TNF-α and IL-6 [50]. Thus, during the tumorigenesis, autophagy can suppress the formation of chronic inflammatory microenvironment by indirectly and directly regulating the production of inflammatory signals.

However, inflammatory response has association with every stage of tumor development and has both tumor-promotive and tumor-suppressive effects. The exact effects of autophagy inhibition-enhanced inflammatory responses in tumor development need to be further exploration and may depend on tumor stages or types.

## Autophagy protects tumor cells survival during tumor progression

### Autophagy promotes oncogene-mediated tumor development

Compare to the role in the process of normal cells tumorization, autophagy seemingly plays opposite role in oncogene-mediated tumorigenesis as a tumor promotor. Except for the difference of tumor type, this strange phenomenon may result from the difference of incipient cells of tumor development. BJ Altman *et al.* found that autophagy deficiency by Atg3 deletion aggravated BCR-Abl-expressing hematopoietic precursor cells death under stresses and prevented BCR-Abl-mediated leukemogenesis [51]. Eileen White laboratory also found that the expression of Ras upregulated basal autophagy, which was required for immortal mouse kidney epithelial cells survival in starvation and in Ras-mediated tumorigenesis [52]. A study about conditional FIP200 knockout mouse model showed that autophagy inhibition retarded MMTV-PyMT-mediated tumorigenesis of mammary epithelial cells by impairing tumor cell survival and proliferation [53].

The process of tumorigenesis involves various activations of oncogenes and inactivations of anti-oncogenes. Strictly speaking, the oncogene-activated cells, which do not need damage/repair cycle and inflammatory microenvironment to trigger continuous proliferation, are not normal cells and have already partly tumorization. In the process of oncogene-mediated tumor development, autophagy perhaps mainly impact on tumor cells and consequently mainly plays a role as tumor promotor.

### Autophagy supports tumor maintenance

In the initiating or rapidly growing stage of tumor development, angiogenesis can not satisfy the great demand of fast-proliferating tumor cells, such as amino acids, oxygen and growth factors. And a series of metabolic stress (including starvation, hypoxia, and ROS accumulation) induce autophagy for survival [23]. Autophagy can digest damaged proteins, organelles and other macromolecules and recycle cytoplasmic materials to balance the demand of nutrients and energy [54]. Hypoxia also induces autophagy for survival, development and metastasis in cancer cells [55,56]. Further study found that HIF-1α, having high expression in hypoxia region of tumors, plays a crucial role in autophagy induction by regulating the expression of its target downstream BNIP3 and BNIP3L [57].

In addition to metabolic stress-activated autophagy, autonomous autophagy also plays crucial role in tumor development. Recently, many studies have shown that several types of tumor cells require autonomous autophagy for tumor growth in normal condition. The Kimmelman laboratory has shown that pancreatic cancers have a distinct dependence on autophagy and require elevated autonomous autophagy for tumor growth. Genetic or pharmacologic inhibition of autophagy leads to increased reactive oxygen species, elevated DNA damage, and a metabolic defect leading to decreased mitochondrial oxidative phosphorylation. In this case, autophagy is likely to provide critical metabolic intermediates that the cells require [58]. Compared to normal cells, Ras-transformed cells possess higher level of autonomous autophagy under basic condition. Several human cancer cell lines bearing activating mutations in Ras commonly have high levels of basal autophagy and are dependent on autophagy for growth [52].

## Autophagy promotes tumor metastasis

As mentioned, autophagy as a metabolic stress adapted mechanism of tumor cells plays a crucial role in tumor development. Currently, many research data show that autophagy also has a significant effect on tumor metastasis. Clinical studies from multiple groups have shown that there is a close relationship between autophagy of cancer cell and tumor metastasis.

LC3B as an autophagosome marker is the most important criterion of estimating the activation and maintenance of autophagy [59]. Rossitza Lazova *et al.* have analyzed nearly 1400 tumors from 20 types of cancer, focusing on correlations between LC3B expression with clinical outcomes in melanoma and breast cancer. Their study found that high level of LC3B was associated with tumor cell proliferation, metastasis, high nuclear grade and worse patient outcome [60]. As mentioned previously, autophagy is required for tumorigenic growth of pancreatic cancers. The study of Satoshi Fujii *et al.* showed significant correlations between the intensity level of LC3 expression in the peripheral area of the pancreatic cancer and tumor size, predominant differentiation, lowest degree of differentiation and blood vessel infiltration [47]. The study of Xiang-Bo Wan *et al.* demonstrated that high expression of Beclin1 protein predicted poorer overall survival and higher occurrence of distant metastasis of nasopharyngeal carcinoma [61]. MI Koukourakis *et al.* have shown that Beclin1 has an important role in growth and metastasis of colorectal cancer [62].

The mechanism of autophagy regulating tumor metastasis remains unclear. Recent study demonstrated that autophagy is critical for HCC cells invasion through the induction of EMT via activating TGF-beta/Smad3-dependent signaling [63]. Besides this, autophagy can promote the survival of the disseminated cells by supporting anoikis resistance and dormancy [64].

### Autophagy supports anoikis resistance and tumor dormancy

It’s crucial for epithelial cells contacting and attaching with the nearby epithelial cells and extracellular matrix (ECM). Lack of normal attachment with their neighbors and ECM, leads to anoikis, which is a type of apoptosis [65]. In order to survive in the circulation and metastasize to a distant organ site, the disseminated epithelial cells need to possess anoikis resistance [66]. The study of Fung *et al.* has shown that the detachment induced autophagy in both nontumorigenic epithelial lines and in primary epithelial cells. ATGs deletion enhanced apoptosis, and reduced clonogenic recovery after anoikis [67]. And many studies have shown that the rapid development of carcinoma tissue needs mass energy for maintaining the proper metabolism. And the lack of energy can induce autophagy in the poverty fields. The ongoing activated autophagy promotes epithelial cell survival during anoikis, including detached cells harboring anti-apoptotic lesions. Moreover, autophagy in ECM-detached cells may compensate for maintaining the loss of nutrient and energy metabolism [68]. And the complex action of autophagy enhances the metastatic potential of carcinoma cells.

Dormancy is one of the notable hallmarks of carcinoma cells. The dormant carcinoma cells as the disseminated seeds survive in the circulation and the distant organ site. Remarkably, these cells are so difficult to diagnosis and kill that lead to recurrence and metastasis [69,70]. Recent studies show that autophagy may be the survival mechanism of the dormant cells. The study of White DE *et al.* have shown that inhibition of integrin β1 signaling induced and maintained autophagy, and then promoted dormancy in the MMTV–PyMT model of breast cancer. The integrin β1 signaling of disseminated carcinoma cells has usually been ruined. And this impaired signaling transduction system can activate autophagy, and leads to dormancy [71]. Recently Lu *et al.* found a direct interaction between autophagy and dormancy. Aplasia Ras homolog member I (ARH I), a tumor suppressor gene, resulted in autophagic cell death of human ovarian cancer cells *in vitro*. However, within xenograft tumors in mice, multiple factors within the tumor microenvironment switched ARHI-induced autophagy to a mechanism of tumor cell survival, leading to tumor dormancy. ARHI-induced autophagy allowed the disseminated dormant cancer cells survival in the circulation and eventually recurred as micrometastasis. And in this process the ARHI tumor suppressor played a role as a switch [72].

Taken together, autophagy-related proteins were associated with poor outcome and aggressive tumor phenotype. On the one hand, autophagy switches the disseminated cancer cells into dormancy for survival in the circulation and eventually recurs as micrometastasis. On the other hand, autophagy induces anoikis resistance for survival and dissemination in the circulation of cancer cells.

## Conclusion and perspective

**Figure 2:**
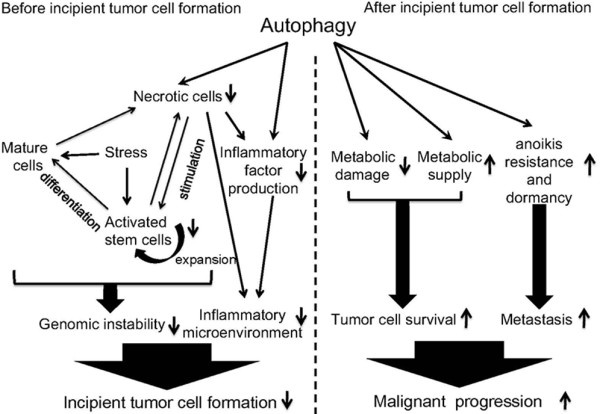
**Protective role of autophagy during early and late stages of tumorigenesis.** Before incipient tumor cell formation, autophagy protects genomic stability through retarding stem cells-involved damage/repair cycle, and inhibits inflammatory microenvironment formation, thus preventing incipient tumor cell formation. After incipient tumor cell formation, autophagy also protects tumor cells survival by decreasing metabolic damage and increasing cellular metabolic supply. Meanwhile, autophagy promotes anoikis resistance and dormancy for providing favorable condition for tumor metastasis. In a word, autophagy protects normal cells or tumor cells during the early or late stage of tumorigenesis, respectively.

Numerous studies suggest that autophagy always is a protector during tumorigenesis, even if it plays dual roles as tumor suppressor and promotor in different stages. Autophagy protects normal cell homeostasis during early stage of tumorigenesis by limiting genome instability via retarding stem cells involved damage/repair cycle, and inhibiting the formation of inflammatory microenvironment. On the other hand, during late stage of tumorigenesis, autophagy protects tumor cells survival by supporting metabolic demand and decreasing metabolic damage. Moreover, autophagy enhances migratory behavior of tumor cells by promoting anoikis resistance and dormancy (Figure 2).

Therefore, the process of tumorigenesis perhaps needs to undergo twice autophagy-associated screening. The normal cells that have lower autophagy capacity due to activation of autophagy-inhibitory pathway are prone to tumorization, and the incipient tumor cells that have more autophagy-bearing capacity or higher autophagy level possibly due to the upregulation of autophagy-promotive pathway are easier to survival in the hash microenvironment and accumulate more mutations to promote malignant progression.

Even if these tremendous autophagy-associated studies have prominently deepened our understanding of the role of autophagy in cancer, there are still so many areas regarding the impact of autophagy on cancer which have not been fully clarified. The study of Yue, W *et al.* indicated the association between autophagy and cancer stem cell (CSC). They found that autophagy inhibition interferes with the maintenance of breast cancer stem-like/progenitor cells [73]. Well, compared to common cancer cell, whether CSC has higher autophagy level or more autophagy-bearing capacity? If it is true, is this ability associated with other properties of CSC, such as higher oncogenicity? Autophagy is a metabolic stress responsor. Is the alteration of autophagy level related to the change of metabolic pattern, from aerobic metabolism to glycolysis, during tumorigenesis? Besides these, basal levels of autophagy in different type of cancer cells are different. Is autophagy a crucial self-protective mechanism for all types of cancer? Although the study of BJ Altman *et al.* showed that hematopoietic precursor cells had low basal level of autophagy and still partly depend on autophagy in the process of BCR-Abl-mediated leukemogenesis [51], this question is still need to further exploration.

The studies for further understanding the mechanism by which autophagy influences on tumorigenesis will provide improvement for autophagy-targeting therapeutic strategies in cancer prevention and treatment.

## Abbreviations

ULK: Unc-51-like kinase; mTOR: Mammalian target of rapamycin; ROS: Reactive oxygen species; PE: Phosphatidylethanolamine; mTORC1: mTOR complex 1; TNF-α: Tumor necrosis factor-alpha; IFN-γ: Interferon-gamma; MAP1S: Microtubule-associated protein 1 small form; DEN: Diethylnitrosamine; PyMT: Ployoma middle T; ECM: Extracellular matrix; ARH I: Aplasia Ras homolog member I.

## Competing interests

The authors declare that they have no competing interests.

## Authors’ contributions

KS and WJD contributed equally to this manuscript. KS, WJD and LXW planned the manuscript outline. KS, WJD, SSZ, NC, SFJ and JRS wrote the draft manuscript, LXW finalized the manuscript. All authors read and approved the final manuscript.
